# A distinct chemokine axis does not account for enrichment of Foxp3^+^ CD4^+^ T cells in carcinogen-induced fibrosarcomas

**DOI:** 10.1111/imm.12430

**Published:** 2015-04-14

**Authors:** Beatrice Ondondo, Emily Colbeck, Emma Jones, Kathryn Smart, Sarah N Lauder, James Hindley, Andrew Godkin, Bernhard Moser, Ann Ager, Awen Gallimore

**Affiliations:** 1 Institute of Infection Immunity and Biochemistry, Cardiff University School of MedicineCardiff, UK

**Keywords:** chemokines, regulatory T cells, tumour immunology

## Abstract

The frequency of CD4^+^ Foxp3^+^ regulatory T (Treg) cells is often significantly increased in the blood of tumour-bearing mice and people with cancer. Moreover, Treg cell frequencies are often higher in tumours compared with blood and lymphoid organs. We wished to determine whether certain chemokines expressed within the tumour mass selectively recruit Treg cells, thereby contributing to their enrichment within the tumour-infiltrating lymphocyte pool. To achieve this goal, the chemokine profile of carcinogen-induced fibrosarcomas was determined, and the chemokine receptor expression profiles of both CD4^+^ Foxp3^−^ and CD4^+^ Foxp3^+^ T cells were compared. These analyses revealed that the tumours are characterized by expression of inflammatory chemokines (CCL2, CCL5, CCL7, CCL8, CCL12, CXCL9, CXCL10 and CX3CL1), reflected by an enrichment of activated Foxp3^−^ and Foxp3^+^ T cells expressing T helper type 1-associated chemokine receptors. Notably, we found that CXCR3^+^ T cells were significantly enriched in the tumours although curiously we found no evidence that CXCR3 was required for their recruitment. Instead, CXCR3 marks a population of activated Foxp3^−^ and Foxp3^+^ T cells, which use multiple and overlapping ligand receptor pairs to guide their migration to tumours. Collectively, these data indicate that enrichment of Foxp3^+^ cells in tumours characterized by expression of inflammatory chemokines, does not occur via a distinct chemokine axis, thus selective chemokine blockade is unlikely to represent a meaningful therapeutic strategy for preventing Treg cell accumulation in tumours.

## Introduction

Tumours are known to employ a number of mechanisms to evade host immune responses. One such mechanism is suppression of otherwise potent T cells,^1^ natural killer cells,^2^ natural killer T cells^3^ and dendritic cells^4^ by CD4^+^ Foxp3^+^ regulatory T (Treg) cells. It has been shown in human tumours and in experimental animal models of cancer that the frequency of Treg cells is significantly increased in peripheral blood, lymph nodes and spleens and that even higher frequencies are found within tumours.^5^–^7^

Several mechanisms that may account for enrichment of Treg cells are reviewed in ref. 8. There is evidence to support a role for transforming growth factor-*β* in promoting their proliferation in tumours and in tumour-draining lymph nodes (TDLN)^9^,^10^ and also for driving conversion of conventional CD4^+^ Foxp3^−^ T cells (Tconv cells) into CD4^+^ Foxp3^+^ Treg cells.^11^ In a previous study whereby the carcinogen methylcholanthrene (MCA) was used to induce fibrosarcomas in mice, we examined the T-cell receptor (TCR) repertoires of Tconv cells and Treg cells and found that the repertoires of tumour-infiltrating Tconv and Treg cells were distinct. This lack of TCR overlap observed between the two populations argues strongly against the hypothesis that Treg cell enrichment in tumours occurs through conversion of Tconv cells into Treg cells.^12^ Another possibility is that intra-tumoural Treg cell enrichment occurs through selective recruitment of Treg cells via tumour-expressed chemokines.^13^–^16^ There is however a general lack of comparative data on the chemokine receptor expression profiles of Tconv versus Treg cells, limiting the understanding of whether a single or multiple chemokine(s) can selectively promote Treg cell recruitment.

In the study described herein, we conducted a broad analysis of chemokine expression by MCA-induced fibrosarcomas and a side-by-side analysis of Foxp3^+^ and Foxp3^−^ CD4^+^ T cells in terms of their phenotype and migratory capacity. The study describes delineation of the chemokine profile of MCA-induced tumours as well as the chemokine receptors expressed by both Tconv and Treg cells. This information was subsequently used to test the hypothesis that the tumour chemokine profile allows for selective accumulation of Treg cells, thereby contributing to immunosuppression within the tumour microenvironment.

## Materials and methods

### Mice

Six- to eight-week-old female C57BL/6 (Thy1.1) mice and Foxp3-GFP transgenic mice, obtained from Professor Alexander Rudensky,^17^ were housed under specific pathogen-free conditions. All experiments were conducted in compliance with UK Home Office regulations.

### Tumour induction

Mice were anaesthetized and injected subcutaneously (in the hind leg) with 400 μg of 3-methylcholanthrene (MCA; Sigma-Aldrich, St Louis, MO) in 100 μl of olive oil. Tumours occurred between 80 and 150 days after injection. Tumour-bearing mice were killed before the tumours reached 1·5 cm in diameter.

### RNA extraction and quantitative RT-PCR

Spleen, lymph node and tumour tissues were snap frozen in liquid nitrogen and stored at −80° until needed. Total RNA was extracted using TRIzol reagent (Invitrogen, Carlsbad, CA) under the manufacturer's specification. The quantity and quality of RNA were determined using the Agilent 2100 bioanalyzer (Agilent Technologies, Santa Clara, CA), and only high-quality samples with an RNA integrity number ≥ 8 were used for quantitative RT-PCR. Five micrograms of total RNA (equivalent to 50 ng RNA per gene) was used for first-strand cDNA synthesis and elimination of contaminating genomic DNA, performed using the RT^2^ First strand kit (SABiosciences, Frederick, MD) as specified by the manufacturer. The cDNA was used in a highly validated quantitative RT-PCR array (RT^2^ Profiler™ PCR array for Mouse inflammatory cytokines and receptors; PAMM-011; SABiosciences) to detect and quantify gene expression levels. Samples were run in a 96-well plate in an ABI 7900HT FAST Block instrument (Applied Biosystems, Foster City, CA) using a two-step cycling programme as follows: step 1, 95° for 10 min; step 2, 95° for 15 seconds, followed by 60° for 1 min; step 2 was repeated for 40 cycles. Data were analysed using the ΔΔC_t_ method. Specific mRNA expression levels for each gene were normalized as a ratio relative to expression of internal control housekeeping genes, namely glucuronidase *β* (*Gusb*), hypoxanthine guanine phosphoribosyl transferase 1 (*Hprt1*), heat-shock protein 90 000 *α* cytosolic class B member 1 (*Hsp90ab1*), glyceraldehyde-3-phosphate dehydrogenase (*Gapdh*) and cytoplasmic *β*-actin (*Actb*).

### Lymphocyte isolation

Spleens and lymph nodes were disrupted by mashing through a 40-μm nylon cell strainer (BD Biosciences, San Jose, CA) using a sterile 2-ml syringe plunger. Tumours were excised and chopped into pieces using scalpel blades. The pieces were then mashed with a syringe plunger and the resulting cell suspension was filtered through 70-μm nylon cell strainers (BD Biosciences). The cell suspensions were then centrifuged before red blood cells lysis using ammonium–chloride–potassium lysis buffer (Gibco, Grand Island, NY).

### Flow cytometry and antibodies

Mononuclear cells isolated from spleens, lymph nodes and tumours were first stained with a dead cell marker (LIVE/DEAD Fixable Aqua stain; Invitrogen) according to the manufacturer's instructions. Cells were then washed and stained for cell surface markers and chemokine receptors. Stained cells were washed, fixed and acquired on a FACS Canto II flow cytometer (BD Biosciences). Data analysis was performed using summit 4.3 software (Dako, Glostrup, Denmark). The antibodies used were as follows: anti-CD4-Pacific Blue antibody (BioLegend, San Diego, CA), goat polyclonal antibody to CCR1 (Santa Cruz Biotechnology, Santa Cruz, CA) followed with phycoerythrin (PE)-conjugated rabbit polyclonal antibody to goat IgG (Abcam, Cambridge, UK), rabbit monoclonal antibody to CCR2 (Abcam) followed with PE-conjugated donkey polyclonal antibody to rabbit IgG (Abcam), anti-CCR4-PE-Cy7 (BioLegend),anti-CCR3-Alexa Fluor 647 (BioLegend), biotin-conjugated anti-CCR5 antibodies followed with streptavidin-PerCP-Cy5.5 (both from eBioscience, San Diego, CA), anti-CXCR3-APC (eBioscience, San Diego, CA), anti-CXCR4-APC (BD Biosciences), rabbit polyclonal antibody to anti-CX_3_CR1 (Abcam) followed with PE-conjugated donkey polyclonal antibody to rabbit IgG (Abcam). Foxp3 expression was detected by GFP fluorescence.

### Chemokine receptor desensitization

For CXCR3 desensitization, splenocytes were treated with 500 nm of CXCL10 for 20 min. To inhibit signalling via chemokine receptors, splenocytes were treated with 25 nm Pertussis toxin for 20 min.

### *In vivo* migration assays

CD4^+^ T cells were isolated from spleens of donor mice by negative isolation with a CD4^+^ T-cell mouse isolation kit II (Miltenyi Biotec, Bergisch Gladbach, Germany) as detailed in the manufacturer's protocol. The purified CD4^+^ T cells were adoptively transferred by intravenous injection and homing observed approximately 24 hr later. Adoptively transferred cells were identified by labelling with fluorescent dyes PKH26 or CFSE and in some instances donor cells were distinguished based on Thy1.2 versus Thy1.1 expression. At the end of the transfer period spleens, lymph nodes and tumours were harvested and the recovered cells were enumerated by flow cytometry. The ratio of Treg cells to Tconv cells in the input fraction of donor cells was compared with that of recovered migrated cells. Antibodies used were: anti-CD90.1 (Thy1.1)- PerCP-Cy5.5 (clone OX-7; BioLegend) and anti-CD90.2 (Thy1.2)-PE (clone 30-H12; BioLegend).

Spleen cells purified from naive wild-type (WT) or CXCR3^−/−^ mice were labelled with the fluorescent dye PKH26 (Sigma, St Louis, MO) or Claret (Sigma) and 2 × 10^6^ cells were injected (100 μl) intravenously. Single cell suspensions were prepared from the spleens, inguinal lymph nodes and tumours approximately 18 hr later and stained with antibodies against CD4. Stained cells were analysed by flow cytometry as described above.

## Results

### MCA-induced fibrosarcomas do not preferentially attract Foxp3^+^ T cells

**Figure 1:**
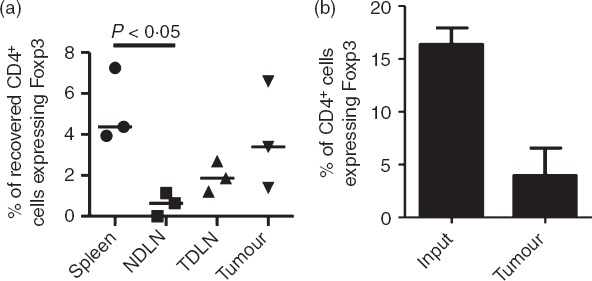
Methylcholanthrene-induced fibrosarcomas do not preferentially attract Foxp3^+^ T cells. In three independent experiments, CD4^+^ T cells isolated from spleens were labelled with CFSE, adoptively transferred into tumour-bearing recipients and 24 hr later, cells from the spleen, non-draining lymph nodes (NDLN), tumour-draining lymph nodes (TDLN) and tumour were analysed by flow cytometry. (a) Frequencies of CD4^+^ Foxp3^+^ T cells among the adoptively transferred cells recovered from spleens, NDLN, TDLN and tumours. Horizontal lines represent the medians. One-way analysis of variance/Dunn's *post-hoc* test were used for statistical analysis. (b) Percentage of CD4^+^ T cells expressing Foxp3 among the input T-cell population and following their recovery from tumours, approximately 24 hr later.

We first wished to determine whether Foxp3^+^ T cells were more likely to migrate to carcinogen-induced fibrosarcomas compared with Foxp3^−^ T cells. To achieve this, splenic T cells were purified and labelled before intravenous injection into tumour-bearing mice. After approximately 24 hr T cells were recovered for analysis by flow cytometry (Fig.1). Although we observed a trend for preferential migration of Treg cells to the tumour and a significant enrichment in the spleen compared with the lymph nodes (Fig.1a) we found that Foxp3^+^ T cells recovered from the tumour were not enriched within the recovered CD4^+^ T-cell population compared with the input population (Fig.1b), thereby indicating that Foxp3^+^ T cells are not more likely than Foxp3^−^ T cells to migrate to the tumour mass.

### Chemokine signature of MCA-induced fibrosarcomas

We next wished to address whether the chemokines attracting Foxp3^+^ and Foxp3^−^ T cells to the fibrosarcomas were the same or distinct in nature. For this purpose, we first identified the candidate chemokines that could facilitate recruitment of CD4^+^ T cells into the tumour mass. A quantitative RT-PCR array was used to analyse the expression of 26 chemokine genes in tissue samples obtained from tumours, spleens and lymph nodes of tumour-bearing mice. To ensure detection of low copy number chemokine genes we used a relatively high amount of total RNA (50 ng/ml) for each gene analysed. Some of the common housekeeping genes are de-regulated in several cancers,^18^ so the extent of variation in the level of expression of five housekeeping genes (*Gusb*, *Hprt1*, *Hsp90ab1*, *Gapdh* and *Actb*) in tumour tissues (compared with spleens and TDLN) was examined to assess their potential as internal controls for normalizing specific chemokine mRNA expression. Indeed as shown in the Supporting information (Fig. S1a), there were significant variations (*P* < 0·0001) in the expression of these genes, with *Hsp90* being expressed at highest levels. While *Gusb* and *Hprt1* were expressed at consistent levels, *Gapdh* and *Actb* showed the least consistency between tumours. We also compared the level of expression in tumour, spleen and TDLN samples derived from the same animal but found no significant variation between tumours and spleen or TDLN (see Supporting information, Fig. S1b). Since *Hprt1* expression showed the least variation among tumours and among tumours, spleen and TDLN, this gene was selected for data normalization.

**Figure 2:**
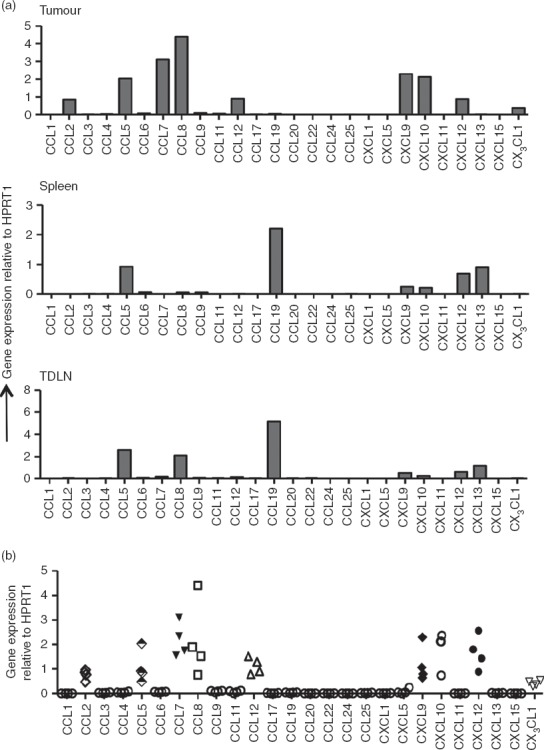
Quantitative RT-PCR for chemokine gene expression. Total RNA was extracted from tumours, spleens and lymph nodes and used for quantitative RT-PCR analysis of various chemokine genes as described in the Materials and methods section. (a) Chemokine gene expression profile of samples from tumour, spleen and tumour-draining lymph nodes (TDLN) of a tumour-bearing mouse. (b) Comparison of chemokine expression levels in tumours from four mice. Gene expression was normalized to *Hprt1* expression as an internal control within each tissue.

As shown in Fig.2(a), MCA-induced tumours showed expression of the inflammatory chemokines CCL2 (MCP-1), CCL5 (RANTES), CCL7 (MCP-3), CCL8 (MCP-2), CCL12 (MCP-5), CXCL9 (MIG), CXCL10 (IP-10), CXCL12 (SDF-1) and CX_3_CL1 (fractalkine). Each of these chemokines was consistently expressed in all of the MCA-induced tumours tested (Fig.2b) and expression of most, namely CCL2, CCL7, CCL8, CCL12 and CX_3_CL1, was detectable only in the tumours and not in spleen and lymphoid tissue. We conclude therefore that these inflammatory chemokines represent the unique chemokine signature of MCA-induced fibrosarcomas. Although expression of CCL5, CXCL9, CXCL10 and CXCL12 were also consistently detected in the tumours, expression of each was also detected in spleen and lymph node.

### Chemokine receptor expression by tumour-infiltrating CD4^+^ T Cells

**Figure 3:**
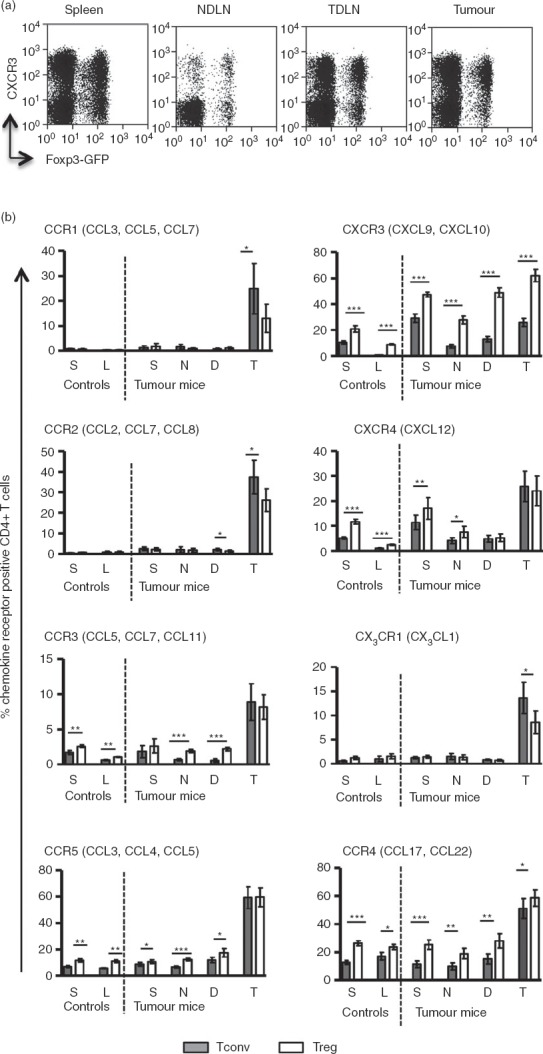
Analysis of chemokine receptor expression by flow cytometry. Single cell suspensions prepared from tumours, spleens and lymph nodes were stained for various chemokine receptors and analysed by flow cytometry. (a) Representative FACS plots showing expression of CXCR3 on conventional T (Tconv) and regulatory T (Treg) cells. (b) Frequency of CD4^+^ T cells expressing chemokine receptors in control (non-tumour-bearing) and tumour bearing mice. Shaded and clear bars represent Tconv and Treg cells, respectively. Letters S, L, N, D and T denote spleen, lymph node, non-draining lymph nodes (NDLN), tumour-draining lymph nodes (TDLN) and tumour, respectively. Paired *t*-test was used for statistical analysis. *P*-value interpretation: ***P ≤ 0·0009; **P = 0·001 to *P* = 0·009; *P = 0·01 to *P* = –0·05. The main chemokines that bind the respective receptors are indicated in brackets.

Treg cells have been shown to express inflammatory chemokine receptors and adhesion molecules suggestive of an enhanced capacity to migrate to inflamed tissues.^19^–^23^ Whether this accounts for the enrichment of Treg cells observed in tumours is not clear as, to our knowledge, few studies have performed a side-by-side analysis of chemokine receptor expression by both tumour-infiltrating Foxp3^+^ and Foxp3^−^ CD4^+^ T cells. Therefore, having established the chemokine profile of the fibrosarcomas under study, we next sought to quantify the proportions of tumour-infiltrating CD4^+^ T cells (both Foxp3^+^ and Foxp3^−^) that expressed the corresponding chemokine receptors for these chemokines. Single cell suspensions prepared from spleens, tumours, non-draining lymph nodes (NDLN) and TDLN were analysed by flow cytometry to determine the expression of chemokine receptors by Foxp3^+^ and Foxp3^−^ T cells. The expression of chemokine receptors binding the tumour signature chemokines defined above was compared on Foxp3^+^ and Foxp3^−^ CD4^+^ T cells (Fig.3). Expression of the inflammatory chemokine receptors, CCR1, CCR2, CCR3, CCR5 and CX_3_CR1, was significantly enriched on both Foxp3^+^ and Foxp3^−^ CD4^+^ T cells within the tumours when compared with spleens and lymph nodes (Fig.3b). These data strongly indicate that inflammatory chemokines CCL2, CCL7, CCL8, CCL12 and CX_3_CL1, all contribute to the intra-tumoural recruitment of CD4^+^ T cells. In addition the data demonstrate that significantly higher proportions of Foxp3^−^ compared with Foxp3^+^ T cells expressed CCR1 (*P* = 0·04), CCR2 (*P* = 0·03) and CX_3_CR1 (*P* = 0·03) whereas no significant differences were observed in expression of CCR3 (*P* = 0·6) or CCR5 (*P* = 0·9) between the two T-cell types. These data suggest that interactions between the chemokine receptors CCR1, CCR2 and CX_3_CR1 with their cognate ligands may favour recruitment of Foxp3^−^ (rather than Foxp3^+^) T cells to the tumours. On the other hand, receptor–ligand interactions for CCR3 and CCR5 would have no bias for either Foxp3^+^ or Foxp3^−^ T cells. Although there is evidence that CCL22 found in ovarian cancers, serves to attract CCR4^+^ Treg cells, we found no evidence for expression of its major ligands CCL22 or CCL17 in the fibrosarcomas described herein.^13^ However, some reports indicate that CCR4 may bind other inflammatory chemokines such as CCL5 and CCL2,^24^ so expression of the receptor was analysed on both tumour-infiltrating Foxp3^−^ and Foxp3^+^ T cells. Although we observed a highly significant difference in proportions of Foxp3^−^ and Foxp3^+^ T cells expressing CCR4 in spleens and lymph nodes, this difference was considerably reduced in the tumour where CCR4 was expressed on a high proportion of both Foxp3^−^ and Foxp3^+^ T cells (Fig.3).

In contrast to these classical inflammatory chemokine receptors, expression of CXCR3, which binds the inflammatory chemokines CXCL9 and CXCL10, was expressed on T cells in all of the compartments analysed (Fig.3b). This is consistent with the detection of CXCL9 and CXCL10 mRNA in spleens, lymph nodes and tumours. There was also a further highly significant enrichment of CXCR3^+^ T cells in the tumour compared with NDLN and TDLN. These data support the hypothesis that CXCR3^+^ T cells (Treg and Tconv) are preferentially recruited to MCA-induced tumours.

**Figure 4:**
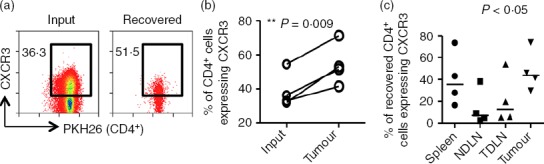
CXCR3^+^ CD4^+^ T cells are enriched in cells recovered from tumours. In four independent experiments, CD4^+^ T cells isolated from spleens of tumour-bearing mice were labelled and adoptively transferred into tumour-bearing recipients for 24 hr. Organs were harvested and cells were stained and analysed by flow cytometry. (a) Representative FACS plots of data comparing the frequency of CD4^+^ T cells expressing CXCR3 among the input transfer fraction and the fraction recovered from the tumour. Numbers indicate the percentage of cells expressing CXCR3. (b) Graph of four independent experiments showing the frequency of CD4^+^ CXCR3^+^ T cells among the input and the fraction recovered from the tumour. Two-tailed, paired *t*-test was used for statistical analysis. (c) The frequency of CD4^+^ CXCR3^+^ T cells among the adoptively transferred cells recovered from spleens, non-draining lymph nodes (NDLN), tumour-draining lymph nodes (TDLN) and tumours was determined by flow cytometry. Horizontal lines represent the medians. One-way analysis of variance/Dunn's *post-hoc* test were used for statistical analysis.

To investigate the enrichment of CXCR3^+^ cells in tumour, splenic CD4^+^ T cells were purified, PKH26-labelled and adoptively transferred into tumour-bearing mice. After 24 hr, tumour-infiltrating PKH26^+^ cells were recovered and stained for CXCR3. When the frequencies of CD4^+^ CXCR3^+^ T cells were compared among the input and migrated fractions recovered from the tumour, a significant enrichment of CXCR3^+^ CD4^+^ T cells was found *P* = 0·009 (Fig.4a,b). This may reflect an enrichment of activated T cells, marked by expression of CXCR3^25^,^26^ and/or preferential migration of CXCR3^+^ T cells to the tumour. Moreover, a significantly higher frequency of CD4^+^ T cells expressing CXCR3 was found in the tumours compared with NDLN (Fig.4c).

### CXCR3 is not required for intra-tumoural recruitment of CD4^+^ T cells

**Figure 5:**
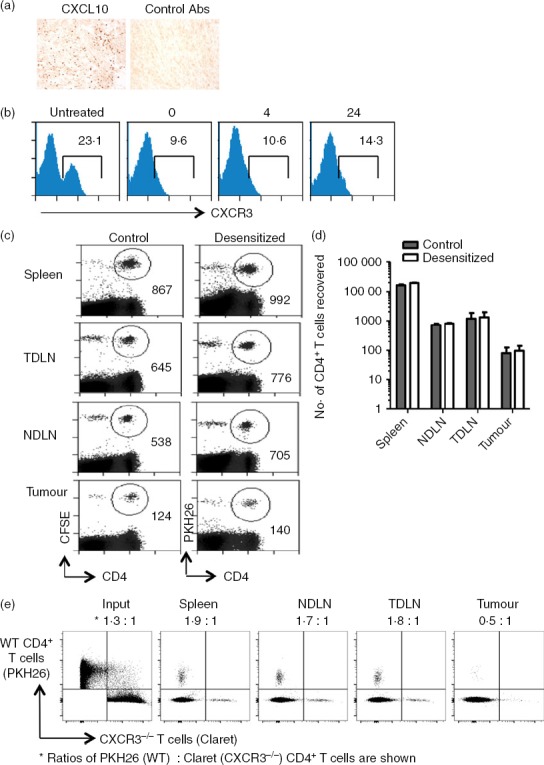
CXCR3 is not required for intra-tumoural recruitment of CD4^+^ T cells. (a) Methylcholanthrene-induced fibrosarcomas were stained by immunohistochemistry for detection of CXCL10 expression. (b). Desensitization of CXCR3 expression by CXCL10 treatment and CXCR3 expression assessed 0, 4 and 24 hr later. (c, d) Mice received 2 × 10^7^ splenic CD4^+^ T cells isolated from spleens of tumour-free Thy1.1 mice, half of which were treated with 500 nm CXCL10 and then labelled with PKH26 (desensitized fraction,). The other half was left untreated (control) and labelled with CFSE. After 24 hr, spleens, non-draining lymph nodes (NDLN), tumour-draining lymph nodes (TDLN) and tumours were harvested, stained and analysed by flow cytometry. (c) Representative plots showing numbers of cells recovered. (d) Absolute numbers of cells recovered from each organ. Results are representative of two independent experiments. (e). PKH26-labelled wild-type (WT) and Claret-labelled CXCR3^−/−^ spleen cells were injected intravenously into a tumour-bearing mouse. Around 18 hr later, the spleen, TDLN, NDLN and tumour were harvested and single cell suspensions were stained with CD4-specific antibodies. Lymphocytes were gated using a live/dead marker and proportions of dye-labelled CD4^+^ cells in each of the compartments are shown.

Based on the observation that CXCR3^+^ T cells are significantly enriched in MCA-induced tumours, we postulated that the CXCR3-binding chemokines (such as CXCL10) were likely candidates for recruitment of T cells to the fibrosarcomas. Indeed immunohistochemical analyses confirmed the presence of CXCL10 within the tumour mass (Fig.5a). To determine whether CXCR3 was required for intra-tumoural recruitment of CD4^+^ T cells we tested whether CXCR3-desensitization through pre-incubation with CXCL10 would interfere with the ability of adoptively transferred T cells to access the tumour. Despite down-regulation of CXCR3 for several hours post-desensitization, no difference in migration to the tumours was observed when compared with the untreated cells (Fig.5b–d), suggesting that although CXCR3^+^ T cells are enriched within tumour immigrants, CXCR3-expression is not required for migration to occur.

To confirm that CXCR3 expression does not promote T-cell migration to the MCA-induced tumours, we performed adoptive transfer experiments where spleen cells purified from WT and CXCR3^−/−^ mice were labelled with the fluorescent dyes, PKH26 and Cellvue Claret, respectively. The labelled cell populations were mixed and injected intravenously into a tumour-bearing mouse. After 18 hr, T cells were recovered from the spleen, tumour and draining (inguinal) and non-draining (contralateral inguinal) lymph nodes (Fig.5e). At this time-point, we found a trend for a small increase in the proportion of WT cells in the spleen and both draining and non-draining lymph nodes relative to the input population. In contrast, most labelled CD4^+^ T cells recovered from the tumour were from CXCR3^−/−^ animals. In a second experiment, where the input ratio of WT : CXCR3^−/−^ CD4^+^ T cells was 1·2 : 1, we again saw a trend for an increase in the proportion of WT cells in the spleen, draining and non-draining lymph nodes relative to the input population (1·3 : 1, 2 : 1 and 2 : 1, respectively) but a small increase in CXCR3^−/−^ CD4^+^ T cells in the tumour (0·9 : 1). Overall, the results obtained in the adoptive transfer experiments confirm that the absence of CXCR3 does not impair CD4^+^ T-cell migration to MCA-induced tumours, and implies that the significant enrichment of CXCR3^+^ CD4^+^ T cells in tumours reflects their activation status and not preferential migration through the CXCR3 receptor.

### Multiple chemokines contribute to intra-tumoural recruitment of T cells

**Figure 6:**
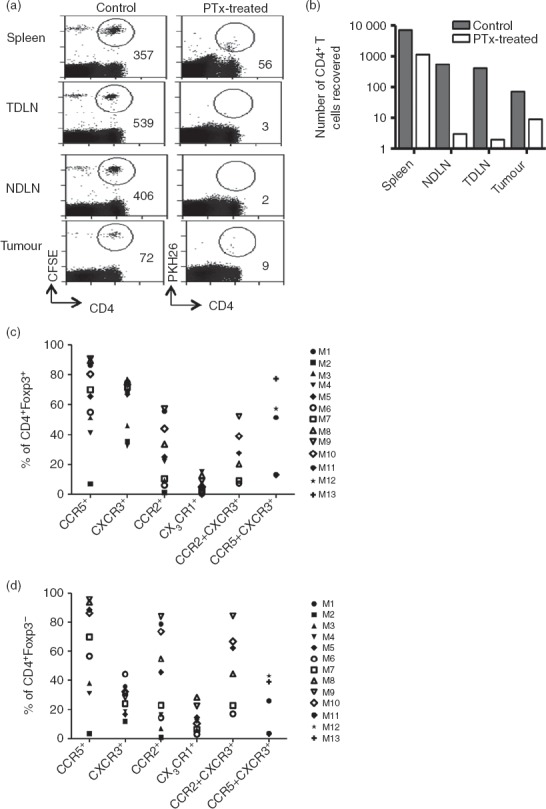
Intra-tumoural recruitment of CD4^+^ T cells is chemokine dependent. Mice received 4 × 10^7^ splenic CD4^+^ T cells isolated from spleens of tumour-free Thy1.1 mice, half of which were treated with 25 nm Pertussis toxin and then labelled with PKH26 (desensitized fraction). The other half were left untreated (control) and labelled with CFSE. After 24 hr, spleen, non-draining lymph nodes (NDLN), tumour-draining lymph nodes (TDLN) and tumour were harvested, stained and analysed by flow cytometry. (a) Plots showing numbers of cells recovered from a representative mouse. (b) Absolute numbers of adoptively transferred cells that were recovered from each organ of a representative mouse. This experiment was carried out on two separate occasions with similar results. (c, d) Single cell suspensions prepared from tumours, spleens and lymph nodes were stained for various chemokine receptors and analysed by flow cytometry. Proportions of tumour-infiltrating regulatory T cells (c) or conventional T cells (d) expressing various chemokine receptors are shown as a percentage of CD4^+^ T cells. Each symbol represents a tumour from an individual mouse, M1–M13.

It is likely that activated T cells express multiple chemokine receptors that facilitate guided migration and specific positioning of the T cells within inflamed tissues including tumours. To test the role of multiple chemokine receptors in T-cell recruitment to tumours *in vivo*, CD4^+^ T cells purified from the spleens of tumour-free mice were treated with Pertussis toxin, an inhibitor of signalling by many chemokine receptors, before adoptive transfer of the cells. An analysis of cells recovered after 24 hr indicated that the Pertussis toxin treatment led to a dramatic reduction in migration of adoptively transferred cells to the tumour and spleens (Fig.6a,b), indicating a major role for chemokine receptors in migration to these sites. As expected, migration to the lymph nodes was completely blocked by Pertussis toxin treatment.

As the data shown in Fig.3 suggest co-expression of CXCR3 and other inflammatory receptors, we looked for co-expression of CXCR3 and other chemokine receptors that are abundantly expressed by tumour-infiltrating Tconv and Treg cells (i.e. CCR5 and CCR2). Data from a group of at least four mice indicated that a high proportion of both Foxp3^+^ and Foxp3^−^ T cells, expressed CXCR3 with CCR2 and/or CCR5 (Fig.6c,d). These data provide evidence for significant overlaps in chemokine receptor expression and indicate that enrichment of CXCR3^+^ T cells most likely occurs through interactions with chemokines binding to co-expressed chemokine receptors. Fundamentally, intra-tumoural recruitment is not a ‘one-chemokine–one-receptor task’ but rather a complex interaction of multiple receptors and chemokines within a tumour-bearing mouse, and as such recruitment-blockade cannot be achieved by targeting a particular chemokine nor can a particular chemokine axis be identified that would serve to block Foxp3^+^ but not Foxp3^−^ CD4^+^ T cells.

## Discussion

In this study we set out to identify mechanisms underpinning the observation that Treg cells are enriched in tumours. Such an observation has been made using numerous mouse models and through the study of patients with cancer. The aim of our work was to investigate within the context of a carcinogen-induced tumour, the relative contribution of chemokine-mediated recruitment to the Treg-enrichment process and to determine whether Foxp3^+^ and Foxp3^−^ CD4^+^ T cells use the same or different chemokines for migration to tumours, in this case MCA-induced fibrosarcomas.

Analysis of the MCA-induced fibrosarcomas revealed a tumour-specific inflammatory chemokine signature and correspondingly, intra-tumoural enrichment of T cells expressing the reciprocal chemokine receptors. This enrichment, in the case of both Foxp3^−^ and Foxp3^+^ CD4^+^ T cells, was highly significant compared with the spleen and lymph nodes, pointing to a continuous chemokine-mediated recruitment of these T cells to the tumours, a conclusion supported by the observation that migration of adoptively transferred T cells to tumours was inhibited by administration of Pertussis toxin. For the most part, we found either no significant difference in the proportions of Foxp3^−^ and Foxp3^+^ CD4^+^ T cells expressing a particular chemokine receptor (CCR3 and CCR5) or, in the case of some chemokine receptors, we found a significant difference in favour of Foxp3^−^ CD4^+^ T cells (CCR1, CCR2 and CX_3_CR1); findings that are compatible with our observation that following adoptive transfer there was no evidence for preferential migration of Foxp3^+^ T cells to the tumour mass.

In the case of CXCR3, although we found that a greater proportion of Foxp3^+^ T cells, compared with Foxp3^–^ T cells, expressed CXCR3 in tumours, CXCR3 was more prevalent on both cell types when comparing tumours to lymph nodes. CXCR3 is a T helper type-1-associated chemokine receptor involved in directing T cells to sites of type 1 inflammation, which is compatible with the chemokine profile of the tumours described herein (ie CCL2, CCL5, CCL7, CCL8, CCL12, CX_3_CR1 and CXCL10).^27^,^28^ A similar finding has been reported previously for human ovarian carcinomas, where CXCR3^+^ T cells represented the majority of Foxp3^+^ and Foxp3^−^ T cells within the tumour mass.^29^ The authors of the study postulated that CXCR3^+^ T cells accumulate in response to type 1 inflammation thereby serving to suppress effector T cells of a T helper type 1 orientation, i.e. those that would otherwise be involved in effective anti-tumour immunity.

In terms of the Foxp3^+^ tumour-infiltrating T-cell population, detailed phenotypic characterization revealed that the majority of these displayed an activated effector memory phenotype, i.e. up-regulating CD44, down-regulating both CCR7 and CD62L and expressing CD103.^30^ Treg cells exhibiting this phenotype have been described as ‘inflammation-seeking’, highly suppressive and expressing high levels of mRNA for inflammatory chemokine receptors CCR2, CCR5 and CXCR3,^31^ consistent with our observations that CXCR3 is co-expressed with these receptors. In the case of the Foxp3^−^ CD4^+^ T-cell population, the tumour-infiltrating T-cell population is far more heterogeneous. Although CXCR3^+^ cells were enriched in this population compared with spleen and particularly lymph nodes, this conventional T-cell population comprises both activated and naive T cells. As we have described previously, naive T cells may access MCA-induced fibrosarcomas via unconventional routes such as aberrantly formed blood vessels and lymphatics.^30^

Curiously, although CXCR3^+^ T cells were enriched compared with CXCR3^−^ T cells in the fibrosarcomas, we found no evidence for a requirement for CXCR3 in the migration process. As mentioned above, we found that CXCR3 is co-expressed with other major receptors (CCR5 and CCR2), suggesting that enrichment of CXCR3^+^ cells most likely also occurs via recruitment by the inflammatory chemokines CCL2, CCL5, CCL7 and CCL8. Hence, enrichment of T cells expressing certain chemokine receptors at a given site may not in itself identify a chemokine axis through which a cell is recruited, but rather reflects the combination of chemokine receptors expressed by that cell.

Other studies in animal models and humans have implied that there is specific recruitment of Treg cells to tumours via certain chemokines. It was recently demonstrated that production of CCL21 in tumours serves to recruit CCR7^+^ Treg cells, thereby promoting tumour growth.^15^ This is not the case for the MCA-induced fibrosarcomas described herein. Although CCL21 can readily be detected within these tumours, few CCR7^+^ Foxp3^+^ T cells are found within the tumours compared with a relatively large proportion of CCR7^+^ Foxp3^−^ T cells.^15^ Although the implications of these findings are yet to be elucidated, the data certainly argue against CCL21-CCR7 blockade as a means of preventing Treg cell enrichment at least in the carcinogen-induced tumours under study here. Other chemokines have been implicated as important for recruitment of Treg cells in different tumour models/cancers.^8^ A role for CCL20-mediated recruitment of CCR6^+^ Treg cells has been demonstrated in a mouse model of colorectal cancer, CCL17 and CCL22 influence recruitment of CCR4^+^ Treg cells in gastric cancer and ovarian carcinoma, and CCL5 can direct recruitment of CCR5^+^ Treg cells in a murine model of pancreatic cancer.^13^,^14^,^16^,^32^ Collectively these findings point to heterogeneity in chemokine expression between different tumours, which is likely to be influenced not only by the tumour cells themselves but also by the nature of the immune infiltrate (e.g. the phenotype of the tumour-infiltrating macrophages). Moreover, the results of some studies indicate that chemokine blockade may reduce both the infiltration of Treg cells and conventional anti-tumour T cells and furthermore, reduce the anti-tumour efficacy of these anti-tumour effector cells.^8^ The results observed in this study support that conclusion as the Treg and Tconv cells display very similar profiles of chemokine receptors. Overall therefore, in the case of many tumours it is unlikely that chemokine blockade will prove effective at promoting tumour immunity through consistent and selective reduction in intra-tumoural Treg cell numbers.

How therefore could Treg cells be targeted for the purpose of cancer immunotherapy? The results of clinical trials using low-dose cyclophosphamide indicate that modulation of Treg cell frequencies in this way can result in enhanced endogenous anti-tumour responses and responses induced by vaccination. Whether such an approach will prove useful for controlling tumour progression is yet to be seen. Knowledge of how Treg cells become enriched in tumours should prove useful. Studies of carcinogen-induced tumours and mouse models of glioblastoma support the notion that proliferation of naturally occurring T cells accounts for the enrichment of Treg cells within tumours.^33^,^34^ A better understanding of factors underpinning this process of preferential proliferation may reveal new and more effective targets for manipulating Treg cell numbers within tumours.

## Abbreviations

Ct: threshold cycle
NDLN: tumour non-draining lymph nodes
TDLN: lymph nodes draining the tumour

## Supporting Information

**Figure S1.** Expression of housekeeping genes.
