# Supplementary figures and images for: A distinct chemokine axis does not account for enrichment of Foxp3+ CD4+ T cells in carcinogen-induced fibrosarcomas
Source: Immunology. 2015 Apr 14;145(1):94–104. doi: 10.1111/imm.12430 (PMC4405327; doi:10.1111/imm.12430)

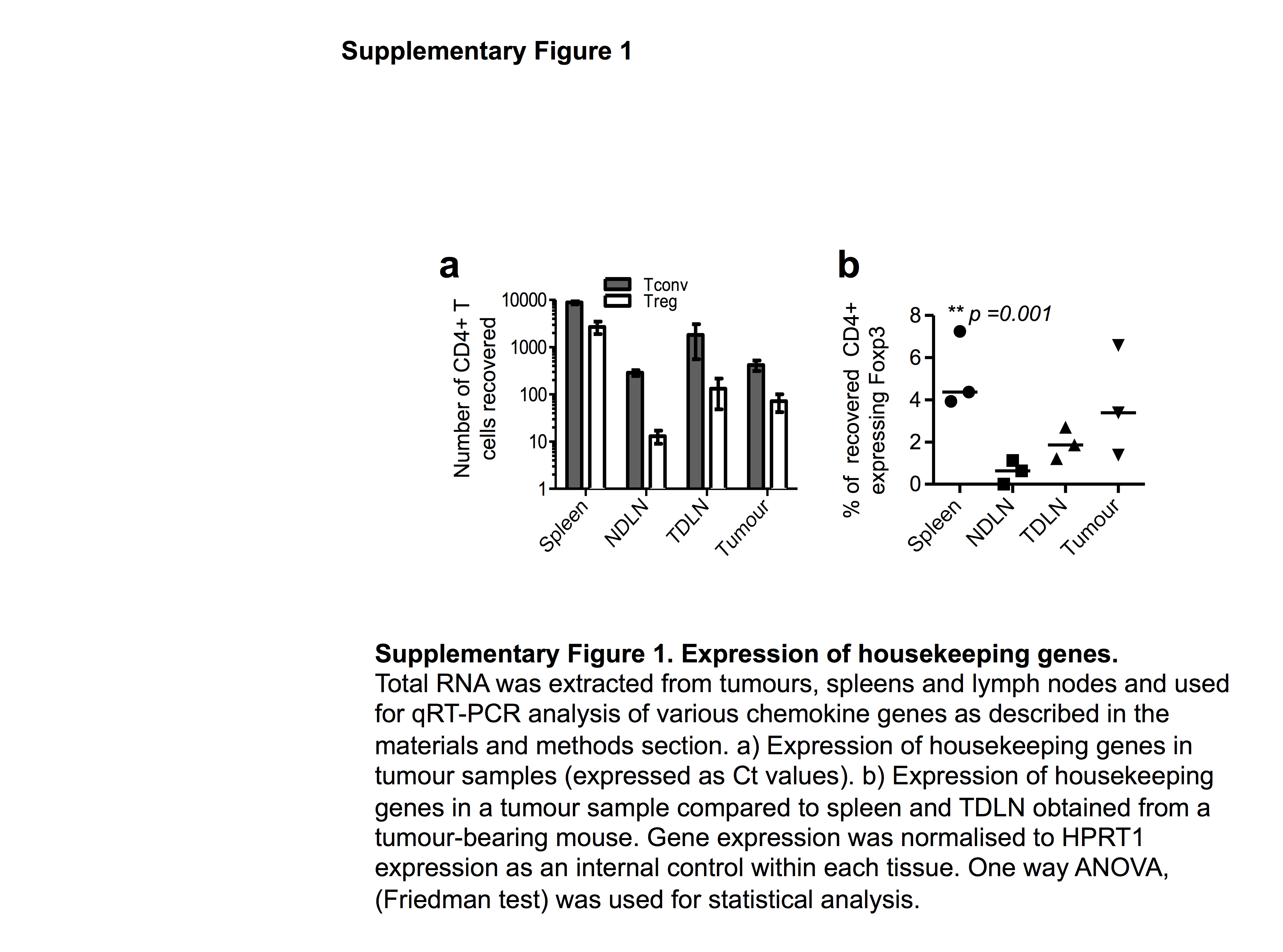

Supplement: Supplementary file 1 [file imm0145-0094-sd1.tif]
